# Supplementary material for: Identification of photoperiod-induced specific miRNAs in the adrenal glands of Sunite sheep (Ovis aries)
Source: Front Vet Sci. 2022 Jul 22;9:888207. doi: 10.3389/fvets.2022.888207 (PMC9354845; doi:10.3389/fvets.2022.888207)
Supplement: Supplementary file 1 [file Data_Sheet_1.ZIP › Supplementary file/miRDeep2 file.zip!/miRDeep2 file/Novel_Structure/SPLP42A3/Novel_45.pdf]

Provisional ID	:	NC_019483.2_37143
Score total	:	18.5
Score for star read(s)	:	3.9
Score for read counts	:	12.4
Score for mfe	:	0.7
Score for randfold	:	1.6
Score for cons. seed	:	
Total read count	:	36
Mature read count	:	34
Loop read count	:	0
Star read count	:	2

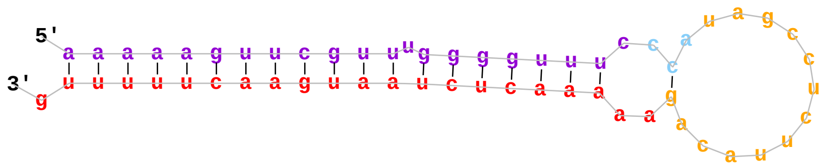

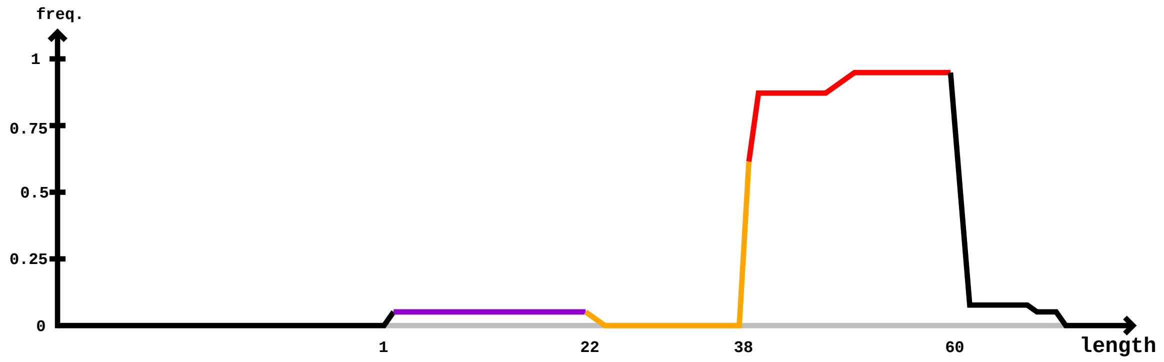

Star   Mature

```
5'- ggacuucuaucuguaaaguauauuggauugaccaaaaaguucguuugggguuucccauagccucuuacagaaaaacucuaaugaacuuuuuggccaacccaauaauagug   -3'    obs
    ggacuucuaucuguaaaguauauuggauugaccaaaaaguucguuugggguuucccauagccucuuacagaaaaacucuaaugaacuuuuuggccaacccaauaauagug          exp
    ....................((((((.(((.((((((((((((((.(((((((..(.............)..))))))))))))))))))))).))).))))))......    reads       mm          sample
    .................................aaaaaguucguuugggUuuuc........................................................    2           1           seq
    ......................................................................aaaaacucAaaugaacuuuuug..................    24          1           seq
    .......................................................................aaaacucAaaugaacuuuuug..................    10          1           seq
    ...............................................................................aaugaacuuuuuggccaaccc..........    1           0           seq
    ................................................................................augaacuuuuuggccaacccaaA.......    1           1           seq
    .................................................................................ugaacuuuuuggccaacccaau.......    1           0           seq
```
